# Survival rate and perioperative data of patients who have undergone hemipelvectomy: a retrospective case series

**DOI:** 10.1186/s12957-016-1001-7

**Published:** 2016-10-07

**Authors:** Alfredo Guilherme Haack Couto, Bruno Araújo, Roberto André Torres de Vasconcelos, Marcos José Renni, Clóvis Orlando Da Fonseca, Ismar Lima Cavalcanti

**Affiliations:** 1 Department of Anesthesiology, Universidade Federal Fluminense, Niterói, Brazil; 2 Department of Anesthesiology, Instituto Nacional de Câncer José de Alencar Gomes da Silva, Rio de Janeiro, Brazil; 3 Department of Surgical Oncology, Instituto Nacional de Câncer José de Alencar Gomes da Silva, Rio de Janeiro, Brazil; 4 Hospital do Câncer II, Instituto Nacional de Câncer José de Alencar Gomes da Silva, Rio de Janeiro, Brazil; 5 Department of Neurosurgery, Universidade Federal Fluminense, Niterói, Brazil; 6 Instituto Nacional de Câncer José de Alencar Gomes da Silva, Rio de Janeiro, Brazil

**Keywords:** Anesthesia, Perioperative care, Cancer, Hemipelvectomy, Survival

## Abstract

**Background:**

Hemipelvectomy is a major orthopedic surgical procedure indicated in specific situations. Although many studies discuss surgical techniques for hemipelvectomy, few studies have presented survival data, especially in underdeveloped countries. Additionally, there is limited information on anesthesia for orthopedic oncologic surgeries. The primary aim of this study was to determine the survival rate after hemipelvectomy, and the secondary aims were to evaluate anesthesia and perioperative care associated with hemipelvectomy and determine the influence of the surgical technique (external hemipelvectomy [amputation] or internal hemipelvectomy [limb sparing surgery]) on anesthesia and perioperative care in Brazil.

**Methods:**

This retrospective case series collected data from 35 adult patients who underwent hemipelvectomy between 2000 and 2013. Survival rates after surgery were determined, and group comparisons were performed using the Kaplan–Meier method and the log-rank test. Mantel–Cox test and multiple linear regression analysis with stepwise forward selection were performed for univariate and multivariate analyses, respectively.

**Results:**

Mean survival time was 32.8 ± 4.6 months and 5-year survival rate was 27 %. Of the 35 patients, 23 patients (65.7 %) underwent external hemipelvectomy and 12 patients (34.3 %) underwent internal hemipelvectomy. The survival rate was significantly higher in patients with bone tumors than in those with soft tissue sarcomas (*P* = 0.024). The 5-year cumulative probability of survival was significantly lower in patients who underwent external hemipelvectomy than in those who underwent internal hemipelvectomy (*P* = 0.043). In the univariate and multivariate analyses, only advanced disease stage (3 and 4) was identified as a significant independent predictor of reduced survival (*P* = 0.0003). Balanced general anesthesia combined with epidural block was the most frequent anesthesia technique. Median intraoperative crystalloid volume and red blood cell transfusions were 3500 mL and 2 units, respectively.

**Conclusions:**

Overall mean survival time after hemipelvectomy was 32.8 months. Advanced disease stage might be independently associated with reduced survival. Smaller amounts of fluids and transfusions were administered and time to discharge was shorter. Acute and chronic pain as well as wound complications are still important challenges in hemipelvectomy.

## Background

Hemipelvectomy is a major orthopedic surgical procedure indicated in specific situations and regularly performed in highly complex tertiary centers [1, 2]. Primary bone and soft tissue pelvic sarcomas are the main indications for hemipelvectomy [3], which has been used in specific critical pelvic trauma patients [4]. Since it was first performed by Billroth in 1891 [5], perioperative care has been a challenge for all health-care providers involved in this aggressive procedure.

Hemipelvectomy involves the following two different approaches: external approach (with limb amputation) and internal approach (with limb preservation) [5]. In the last few decades, the use of external hemipelvectomy for the treatment of pelvic cancer has declined, and new surgical techniques and efforts for resection with limb preservation (internal hemipelvectomy) and reconstruction have been introduced [6–8].

Survival after hemipelvectomy might be related to several different factors, such as tumor histopathology and size, disease stage, patient physical status, and resection type. In patients with soft tissue tumors, the 5-year survival rate might be as low as 10 %. Large tumors and bone and vascular involvement might be indicators of poor survival [8]. For bone tumor resection, the 5-year survival rate can be as high as 100 %, depending on the resection type [9]. A large previous series reported a survival rate of 50 % after hemipelvectomy [10].

With regard to hemipelvectomy, anesthesia and perioperative care may be considered challenging because of extensive tissue trauma related to the surgery, the use of preoperative adjuvant chemotherapy and radiotherapy, significant blood and fluid loss, bleeding disorders, and intense postoperative pain [11]. In most cases, major neurovascular dissection, large bone resection and reconstruction, and skin flap rotation are needed [2]. Although many studies have discussed surgical techniques for hemipelvectomy, few studies [6, 7, 12] have presented survival data, especially in underdeveloped countries [9]. Additionally, there is limited information on anesthesia for orthopedic oncologic surgeries [2]. We found only one paper that presented anesthetic and perioperative data specifically for hemipelvectomy. The paper was published in 2007 [11].

The primary aim of this study was to determine the survival rate after hemipelvectomy, and the secondary aims were to evaluate anesthesia and perioperative care associated with hemipelvectomy and determine the influence of the surgical technique (external hemipelvectomy [amputation] or internal hemipelvectomy [limb sparing surgery]) on anesthesia and perioperative care in Brazil.

## Methods

This retrospective case series collected data from the medical records and from the Department of Pathology database of all patients who underwent hemipelvectomy at Instituto Nacional de Câncer, Brazil, between 2000 and 2013. Patients aged under 18 years were excluded from the search. The study was approved by the Research Ethics Committee of Instituto Nacional de Câncer José Alencar Gomes da Silva (reference number 82751/CAAE 05859312.9.0000.5274).

The variables of interest were associated disorders, age, sex, weight, American Society of Anesthesiologists Physical Status classification, tobacco use, preoperative chemotherapy and radiotherapy, tumor size and histology, disease stage, type of procedure, operation time, type of anesthesia, neuroaxial opioid use, amount of crystalloids and colloids infused, perioperative transfusion, and extubation. In addition, postoperative variables (acute and chronic pain, surgical wound, and clinical complications) and hospital length of stay were studied.

As no specific guidelines were described in the database or medical charts, the choice of anesthesia technique as well as transfusion triggers was based on individual criteria of personal experience and subjective team evaluation.

Pain was assessed using the visual analog scale (VAS) and was considered severe if the VAS score was >7 and chronic if it persisted for over 12 weeks. Surgical wound complications included infection, fistula, and dehiscence.

Information regarding follow-up and time to death was retrieved from the Department of Cancer Information at our institution. Patients who showed up in the last doctor appointment were considered alive. All deceased patients were registered in the database of the institution.

External hemipelvectomy was indicated in cases where adequate surgical margin could not be obtained with preservation of a viable and functioning limb. Also, it was indicated in large tumors with vascular involvement and ulcerated and great pelvic invasion. Bone reconstruction was not performed after internal hemipelvectomy as it is not routine in this center.

Pelvic resection in internal hemipelvectomy was classified into the following: iliac (T1), acetabular (T2), pubis or ischium (T3), and sacral (T4) [12]. Combinations of procedures were also performed in association with high femoral resection and/or sacral extension.

Disease staging was performed retrospectively by an orthopedic oncologic surgeon following the American Joint Committee on Cancer (AJCC) [13] for soft tissue sarcomas and the Union for International Cancer Control (UICC) for cases involving the bone.

### Statistical analysis

Median, mean, standard deviation, and range were used to summarize numerical data, and frequency (*n*) and percentage (%) were used for categorical data. The associations between variables were analyzed using the Mann–Whitney *U* test for numerical data and the *χ* ^2^ test or Fisher’s exact text for categorical data, as the Shapiro–Wilk normality test was rejected.

The survival rate after surgery was calculated using the Kaplan–Meier method, and the log-rank test was used for comparisons. Survival curves compared groups dichotomized by age (under 50 vs. 50 years or more), tumors (bone vs. soft tissue), and type of surgery (internal vs. external).

To identify factors that could independently influence survival, univariate analysis was performed with Mantel–Cox regression for each variable. The candidate variables included sex, age, preoperative radiotherapy and chemotherapy, transfusion, intraoperative fluid volume, and disease stage. Multivariate analysis was performed using multiple linear regression with forward selection.

All analyses were performed using SAS® System 6.11 (SAS Institute, Inc., Cary, NC). A *P* value <0.05 was considered significant.

## Results

Thirty-five cases were selected for analysis after database searches.

The preoperative data are presented in Table 1. Ambulatory anesthesia preoperative evaluation was performed 2–3 weeks before hemipelvectomy in 12 patients, and 23 patients were visited by an anesthesiologist before surgery after hospital admission. Thirty-three procedures were elective and 2 were considered emergency owing to tumor bleeding.

**Table 1:** Preoperative data

Variable	Value
Age, years (median and range)	40 (18–82)
Weight, kg (mean and range)	69.1 (43–106)
Male/female (*n*)	24:11
Preoperative radiotherapy (%)	28.6
Preoperative chemotherapy (%)	17.1
ASA (*n*)	
1	14
2	13
3	8
Tobacco use (*n*)	15
Arterial hypertension (*n*)	9
Diabetes (*n*)	2
Coronary disease (*n*)	1
Asthma (*n*)	1
Chronic renal failure (*n*)	1
Gastroesophageal reflux disease (*n*)	1
Pulmonary metastasis (*n*)	4
Depression (*n*)	2

*ASA* American Society of Anesthesiologists Physical Status Classification

Twelve patients presented soft tissue tumors and 23 had primary bone tumors. Classic chondrosarcoma was the most frequent case (15 cases, 1 recurrence). Two chondrosarcomas were classified as mesenchymal (bone, 1; soft tissue [extra-skeletal], 1). Among the osteosarcomas, 1 was fibroblastic, 1 was chondroblastic, 1 was small cell, and 3 were classic.

The other sarcomas included 1 clear cell, 2 pleomorphic, 1 high-grade, 1 undifferentiated, and 1 fibrosarcoma. Of these 6 cases, 4 were recurrent. Malignant fibrous histiocytoma was pleomorphic in 2 cases (1 recurrent) and myxoid in 1 case. There were 2 cases of synovial cell sarcoma and 1 giant cell tumor.

Of the 12 cases of soft tissue tumors, 5 were stage 3, 2 were stage 4, and 5 were local recurrences. Of the 23 bone tumors, 13 were stage 2b, 8 were stage 1b, 1 was stage 3, and 1 was local recurrence. Among the 12 patients with soft tissue tumors, 9 (75 %) had tumors >10 cm; and among the patients with bone tumors, 19 (82.6 %) had tumors >8 cm.

The surgical procedures are presented in Table 2.

**Table 2:** Surgical procedures

Type	*N*
Internal type I	5
Internal type I with sacral extension	1
Internal type II	2
Internal type III	1
Internal types II and III	1
Internal types I, II, and III with high femoral resection	2
External	23

**Fig. 1:**
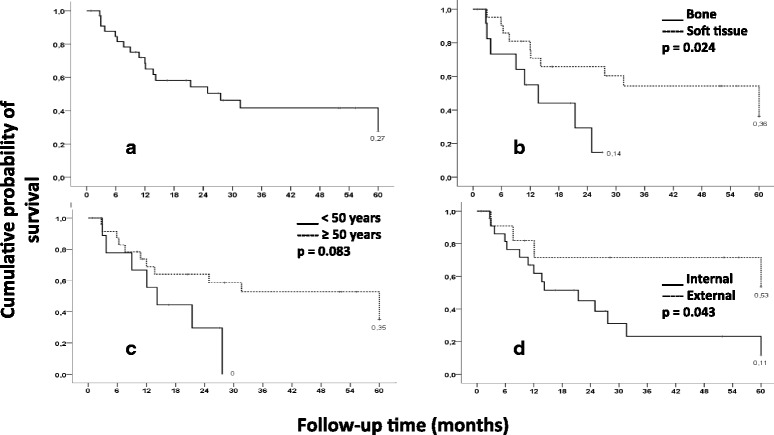
Survival rate after surgery (*n* = 29). **a** Overall survival rate. **b** Survival rate of patients with bone tumors and those with soft tissue tumors. **c** Survival rate of patients aged <50 and those aged ≥50. **d** Survival rate of patients who underwent internal hemipelvectomy and those who underwent external hemipelvectomy

The 5-year survival rates after surgery are presented in Fig. 1.

The mean survival time was 32.8 ± 4.6 months, and the survival rate after 5 years was 27 %. The survival rate was higher among patients with bone tumors than among those with soft tissue sarcomas (*P* = 0.024). Additionally, the cumulative probability of survival at 5 years was lower among patients who underwent external hemipelvectomy than among those who underwent internal hemipelvectomy (*P* = 0.043). Internal hemipelvectomy patients presented a survival rate of 57 % in 5 years while external hemipelvectomy patients had only 11 % in the same time interval. However, the survival rate difference was not statistically significant between patients aged <50 at the time of the procedure and those aged ≥50 at the time of the procedure (*P* = 0.083).

In the univariate analysis, only advanced disease stage (3 and 4) was found to be a significant independent factor for reduced survival among the variables tested (*P* = 0.0003, hazard ratio = 6.65, 95 % CI = 2.36–18.7). Additionally, in the multivariate analysis, only advanced disease stage (3 and 4) was found to be an independent risk factor for reduced survival.

Data on perioperative management are presented in Table 3. The median volume of crystalloid infusion was 3500 mL, and 500-mL multiples were used for colloid administration quantification. Twenty patients received red blood cell (RBC) concentrates intraoperatively and 4 patients received other blood products in the operating room (OR). Thirty-three patients underwent tracheal extubation in the OR. In the perioperative period, a high number of patients presented at least one episode of severe hemodynamic instability (23 patients, 65.7 %). All these patients received fluid resuscitation, and vasopressors (infusion, boluses, or both) were required in 12 of these patients (52.17 %). Six patients presented persistent bleeding disorders with associated abnormal coagulation tests.

**Table 3:** Perioperative data

Variable	Value (*n* = 35)
Operating time (min)^a^	200 (90–460)
Type of anesthesia (*n*)	
General plus regional	31
General alone	3
Regional alone	1
Type of general anesthesia (*n*)	
Intravenous	3
Balanced	31
Type of regional anesthesia (*n*)	
Spinal	4
Epidural block	25
Spinal plus epidural block	3
Intraoperative neuroaxial opioids (*n*)	32
Intraoperative use of colloids, 0.5/1/1.5 L (*n*)	12/07/03
Intraoperative crystalloid volume (mL)^a^	3500 (1000–8500)
Intraoperative albumin infusion (*n*)	6
Intraoperative transfusion (*n*)	20
Intraoperative red blood cells (units)^a^	2 (1–6)
Intraoperative use of other blood products (*n*)	
Fresh frozen plasma	3
Cryoprecipitate	1
Platelets	0
Extubation in the operating room (*n*)	33

^a^Median and range

Postoperative data are presented in Table 4. Severe and acute pain after the procedure was noticed in 31.4 % of the cases and 40 % of the patients developed chronic pain. Postoperative transfusion was performed in 17 patients. With regard to postoperative complications, 1 patient developed acute renal failure, 2 had neurological disorders, 1 had arrhythmia, and 9 developed surgical wound complications. Median hospital stay after surgery was 6 days.

**Table 4:** Postoperative data

Variable	Value
Postoperative transfusion (*n*)	17
Severe acute pain after the procedure (VAS > 7) (*n*)	11
Postoperative complications	
Acute renal failure (*n*)	1
Neurological disorders^a^ (*n*)	2
Arrhythmias (*n*)	1
Chronic pain 3 months after surgery (*n*)	14
Surgical wound complications (*n*)	9
Time to discharge (days)^b^	6 (3–27)

*VAS* visual analog scale

^a^Ischemia delirium and/or disorientation

^b^Median and range

Comparisons of perioperative data between external hemipelvectomy and internal hemipelvectomy (without reconstruction) are presented in Table 5. There were no significant differences in perioperative data between external hemipelvectomy and internal hemipelvectomy (without reconstruction), including time to hospital discharge.

**Table 5:** Perioperative data (external hemipelvectomy vs. internal hemipelvectomy)

		Internal (without reconstruction)			External		
Variable	*n*	Median	Range	*n*	Median	Range	*P* value
Age (years)	12	40	32–46	23	45	29–55	0.39
Weight (kg)	12	75	64–81	23	63	52–83	0.15
Operation duration (min)	12	240	140–284	23	195	160–225	0.53
Intraoperative RBC transfusion (units)	6	4	1.8–6.0	14	2	2.0–3.0	0.13
Intraoperative crystalloid infusion (mL)	12	3750	2825–5000	23	3000	2500–4000	0.29
Postoperative RBC transfusion (units)	3	3	1.0–4.0	21	2	1.0–2.0	0.38
Total RBC transfusion (units)	7	4	1.0–8.0	14	3	2.0–4.0	0.4
Hospital stay after surgery (days)	12	6	5.0–7.0	23	6	4.0–8.0	0.68

*RBC* red blood cell

We compared surgical wound complications and the incidence of chronic pain between external hemipelvectomy and internal hemipelvectomy (without reconstruction) and found no significant differences (*P* = 0.095 and *P* = 0.17, respectively). However, external hemipelvectomy was responsible for 8 of the 9 cases of surgical wound complications. The number of patients with intraoperative hemodynamic instability was higher among patients who underwent external hemipelvectomy than among patients who underwent internal hemipelvectomy (*P* = 0.003).

## Discussion

In this study, mean survival time was 32.8 ± 4.6 months. In a previous study, Penna et al. [9] demonstrated a mean survival time of 43 ± 17 months. This difference could be explained by the fact that a large number of patients had an advanced disease stage at the time of surgery in this study.

We found that the chance of survival was higher with internal hemipelvectomy than with external hemipelvectomy. This is consistent with the findings of a previous study [9]. Therefore, if possible, limb preservation surgery is the first choice when hemipelvectomy is considered a possible approach. The fact that external hemipelvectomy is currently performed in specific situations of more advanced disease such as failed neoadjuvant therapy, severe deep infection, sciatic nerve and femoral vessel infiltration, local tumor recurrence, improvement of the resection margin, and as a life-saving or palliative procedure could possibly explain the higher chances of survival in the internal hemipelvectomy group [2].

Although we did not notice any significant statistical difference in survival when patient age (>50 or ≥50) was considered (*P* = 0.083), Mankin et al. [10] demonstrated a higher survival rate after hemipelvectomy in patients aged <50 than in those aged ≥50. Sample size may have contributed to this difference.

Among the variables selected, only advanced disease stage was a significant predictor of reduced survival in both univariate and multivariate analysis (*P* = 0.001), and this finding is consistent with the results of a previous study [10].

All patients with soft tissue tumors died at 60 months after the surgery in this study. However, Appfelstaedt et al. [8] reported a 10 % survival rate at 5 years for curative surgery and 14 % for palliation. On evaluating the data of patients with soft tissue tumors, we found that almost all the patients had an advanced disease stage (grade 3 or 4), and this might explain the reduced survival.

In the present series, 65.7 % of the procedures were external hemipelvectomy, although limb savage surgeries have been reported to be possible in most cases owing to medical advances [14]. Previous studies [9–12] have reported the predominance of internal hemipelvectomy in the treatment of pelvic cancer. This difference may be explained by the large tumor sizes in this study, and it reflects the high number of patients at an advanced disease stage assisted at Instituto Nacional de Câncer José Alencar Gomes da Silva.

Intraoperatively, a median of 2 units (range, 1–6 units) of RBCs were administered. Hemipelvectomy has been shown to be associated with massive bleeding and significant blood and fluid loss [15]. A previous study reported the requirement of massive transfusions [1], and another reported that a median of 7 units (range, 0–44 units) of RBCs were required intraoperatively [11]. Moreover, intraoperatively, a median crystalloid volume of 3500 mL (range, 1000–8500 mL) was infused, and the volume administered was lower in this series than in the study by Molnar et al. [11] (median, 8500 mL; range, 1000–42,000 mL). Additionally, the operation time was shorter than in the study by Molnar et al. [11] (200 vs. 300 min). These differences might be explained by the fact that none of the patients who underwent internal hemipelvectomy had bone reconstruction in this study, while 19 of 49 patients underwent some form of reconstruction in the study by Molnar et al. [11].

Balanced general anesthesia combined with epidural block was the most frequent choice of anesthesia for hemipelvectomy in this series, and this finding is similar to the finding of Molnar et al. [11]. Although some studies suggested that propofol anesthesia was superior to volatile agents in cancer patients [16, 17], other studies do not support this suggestion [18–20]. In a recent review, Heaney et al. [18] stated that there is no conclusive evidence to indicate that one anesthetic agent is better than another agent in cancer patients. Therefore, there is no reason to change the current practice.

We observed that efforts were made to deliver regional anesthetics in every case, and epidural or spinal local anesthetics were delivered in 32 of the 35 patients. Usually, after the surgical plan was discussed, epidural catheters were positioned in the lower thoracic or upper lumbar spine to avoid interference with the surgical field. A previous study reported that neuroaxial opioids were highly effective at reducing postoperative pain [2]. However, as 31.4 % of patients developed severe postoperative acute pain in our study, neuroaxial opioids were not able to provide adequate postoperative analgesia. Nevertheless, Weinbroum [21] reported the superiority of epidural over intravenous patient-controlled analgesia in orthopedic oncologic patients, and its use is recommended.

Chronic pain was an important postoperative complication. A total of 14 patients experienced this complication, and among the 23 patients who underwent external hemipelvectomy, 11 had persistent pain. Persistent postsurgical pain (pain that lasts for more than 1–2 months after surgery) has been reported to be present in more than 30 % of patients after procedures, such as amputations, and its recognition is increasing [22]. Studies have shown that up to 90 % of patients may experience phantom pain after hemipelvectomy-associated amputation, and although the mechanisms of pain after this procedure are not fully understood, the pain can be debilitating and may impair rehabilitation and quality of life [22, 23]. Neurotoxic chemotherapy, moderate-to-severe postoperative pain, anxiety, younger age (adults), radiation therapy in the operated area, and preoperative pain (moderate to severe) for more than 1 month were previously identified as risk factors for the development of chronic postsurgical pain [24, 25], and all or at least some of these factors may be present in hemipelvectomy patients.

In this study, 25,7 % of cases developed surgical wound complications that included infection, fistula, and dehiscence. Previous studies by Higinbotham et al. [26] and Apffelstaedt et al. [27, 28] reported wound complication incidences of 75 and 47 %, respectively. Beck et al. [22] reported a wound complication incidence of only 4 %. Our findings are somewhat similar to the findings of these previous studies.

The median postoperative length of hospital stay was 6 days (range, 3–27 days), and there was no difference between patients who underwent external hemipelvectomy and those who underwent internal hemipelvectomy. The length of time was shorter than in the study by Beck et al. [22] (14 days for internal and 20 for external hemipelvectomy) and by Molnar et al. [11] (14 days). Team efforts, institutional experience, and a low incidence of clinical complications may explain the earlier hospital discharge in our study.

This study did not focus on rehabilitation data. As a routine, all efforts were made to deliver early rehabilitation. Main goals were to sit in the first postoperative day and stand up in the fourth and, at discharge, patients were scheduled for ambulatory physiotherapy. Amputated patients received focus on wound edges care and preparation for prostheses. Internal hemipelvectomy patients received ambulatory ambulation training, corporal balance, and other mobility training such as gait aid.

A retrospective case series approach is useful to study rare diseases and infrequent procedures. However, several limitations are associated with this approach. This study was performed at a single center, and this might have resulted in bias. The large number of patients with advanced disease stage, especially in the soft tissue sarcoma group, may have contributed to low sample survival as well as worse outcome in external hemipelvectomy. Reports from different centers are important to contribute to increased knowledge about the outcomes of such aggressive and potentially critical procedures.

## Conclusions

In conclusion, hemipelvectomy is an aggressive procedure and despite advances in surgical and perioperative management, the overall mean survival time after surgery may be considered low (32.8 months in the present series). Advanced disease stage might be independently associated with reduced survival and although considerably smaller amounts of fluids and transfusions were administered and time to discharge was shorter, acute and chronic pain as well as wound complications are still important challenges in hemipelvectomy.

## Abbreviations

ASA: American Society of Anesthesiologists
CI: confidence interval
OR: operating room
RBC: red blood cell
VAS: visual analog scale
